# New approaches for extraction and determination of betaine from *Beta vulgaris* samples by hydrophilic interaction liquid chromatography-tandem mass spectrometry

**DOI:** 10.1007/s00216-017-0461-0

**Published:** 2017-06-29

**Authors:** Luca Rivoira, Sylwia Studzińska, Malgorzata Szultka-Młyńska, Maria Concetta Bruzzoniti, Bogusław Buszewski

**Affiliations:** 1 Department of Chemistry, University of Torino, via P. Giuria 5, 10125 Torino, Italy; 2 Department of Environmental Chemistry and Bioanalytics, Nicolaus Copernicus University, ul. Gagarina 7, 87-100 Toruń, Poland; 3 Center for Modern Interdisciplinary Technologies, Nicolaus Copernicus University, Wilenska 4, 87-100 Torun, Poland

**Keywords:** Betaine, *Beta vulgaris*, ASE, QuEChERS, SPE, HILIC-MS/MS

## Abstract

Betaine is one of most studied biologically active compounds, due its role in the main biological processes. Although it may be found in several plants and roots, such as the *Beta vulgaris* family, present in typical diets, just a few analytical methods have been developed for its extraction from roots. A new, quick and effective procedure for the isolation and determination of betaine from two different varieties of *B. vulgaris* (red and gold) is presented. For betaine extraction, an accelerated solvent extraction (ASE) was coupled with solid-phase extraction. For betaine determination, a separation method based on hydrophilic interaction chromatography coupled with tandem mass spectrometry was optimized for a sensible detection of betaine by means of experimental design. Recoveries were about 93%, with RSD <5%, for both the matrices, without evidence of interfering species. The total content of betaine in extracts of various parts of plants (juice, peel, root) have been determined, obtaining concentrations in the range 3000–4000 mg/L for the juice and in the range 2–5 mg/g for the pulp and for the peel. The *B. vulgaris* gold species exhibited a higher concentration of betaine, compared to the red variety. Additionally, a micro extraction by packed sorbent technique and a modified quick, easy, cheap, rugged and safe (QuEChERS) procedure, were also tested and compared. Despite the lower recoveries of the latter, with respect to the ASE/SPE procedure (75–89%, RSD <1.5%), the ease of the method, which can be applied without the SPE purification procedure, can represent a positive improvement.

**Graphical abstract:**
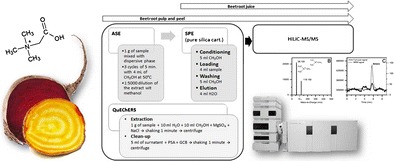
Determination of betaine from *Beta vulgaris* samples.

Determination of betaine from *Beta vulgaris* samples.

## Introduction

**Fig. 1:**
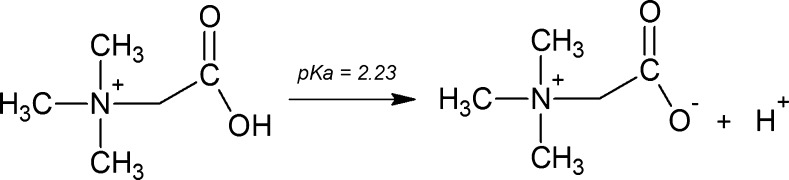
Acid dissociation of betaine

Betaine (trimethylglycine, glycine betaine, lycine, or oxyneurine) is a zwitterionic quaternary ammonium compound, discovered in the nineteenth century in the juice of sugar beets. Since betaine is characterized by a pKa value of 2.33 [1] (Fig. 1), at environmental and physiological pH values, the molecule is present as a zwitterion. The small distance between the ionized groups leads to a heterogeneous solvation shell which is influenced by both the hydrophilicity of the carboxylate group and the hydrophobicity of the methyl groups in the ammonium moiety [2]. Therefore, betaine is a high polar compound (log *P* = −4.49), fully soluble in water and in methanol.

Betaine is an oxidative derivative of choline. In the human body, it could be introduced by the diet, directly or from dietary choline [3].

Among the different, well documented roles of betaine, the following ones are the most relevant: (i) organic osmolyte, (ii) methyl donor for the remethylation of homocysteine, (iii) protector against alcohol-induced liver injury and (iv) biologically important in cancer development [4, 5].

Betaine is a promising agent that attenuates homocysteine rise after meals. Therefore, a diet rich in betaine (or choline) might benefit cardiovascular health through its homocysteine-lowering effects. However, some studies show how high betaine intakes could increase serum lipid concentrations, which of course increases the risk of cardiovascular disease.

As an organic osmolyte, betaine could be found in several plants and roots, which accumulate this highly soluble compound in response to water stresses. Among them, roots from the *Beta vulgaris* varieties (Chenopodiaceae family), which are present in typical food diets, are rich in this compound. One of the most well-known and consumed beetroots is the red *B. vulgaris.* Another diffuse variety is the golden (or gold) one, which has recently been investigated also as source of dyes, due to the high demand of yellow dyes [6].

Betaine extraction from food samples is mainly performed by liquid-liquid (LL) and solid-liquid extractions (SLE), depending on the nature of the sample. Methanol, dichloromethane and chloroform, sometimes partially mixed with water, were used as solvents for cold extraction [7, 8], while methanolic KOH was used together with a boiling Goldfisch apparatus [9]. High-performance liquid chromatography (HPLC) coupled with tandem mass spectrometry (MS/MS) detection [7], UV (using pre- or post-derivatization) [10] and light scattering detection (LSD) [8] were used for the analytical determination. Ion chromatography in the non-suppressed conductivity mode was also employed for analysis [11]. These methods, which were applied for the determination of betaine in feed additives, fruits, seeds or plasma samples, are characterized by limits of detection (LODs) which depend mainly upon the detection mode and the extraction technique used. For example, LODs ranging from 2 to 5 mg/kg were obtained using SLE extraction coupled with HPLC-MS/MS and non-suppressed ion chromatography (IC), respectively. Higher (150 mg/kg) limits were achieved by HPLC-LSD methods [8].

Beside the fair LODs, the above-mentioned extraction techniques are all non-selective for betaine and consequently co-extracted interfering species, affect the sensitivity of the technique. Accordingly, an enhancement in the selectivity of the extraction procedure is desirable in order to improve the detection limits of the entire method.

In light of the above-mentioned considerations, the aim of the present work was the optimization of a quick, easy and selective method for the determination of betaine in food samples. Recently, accelerated solvent extraction (ASE) has been proposed for the extraction of target polar compounds from water-rich food samples [12, 13]. For the first time, ASE was here coupled with solid-phase extraction (SPE) or micro extraction by packed sorbent (MEPS) for the extraction of betaine from *B. vulgaris* samples. Betaine was quantified by hydrophilic interaction chromatography coupled with tandem mass spectrometry (HILIC-MS/MS): the optimization of response, as a function of detector parameters, by experimental design allowed us to obtain improved LODs. Moreover, for the first time, a quick, easy, cheap, rugged and safe (QuEChERS) procedure was successfully optimized through the evaluation of different dispersive-SPE (d-SPE) sorbents and applied for a quick and easy isolation of betaine. Finally, the ASE/SPE method, previously optimized, was applied to the red and gold varieties, determining the distribution of betaine in each portion of the beetroots (peel, pulp and juice) and between the species considered.

## Experimental section

### Chemicals

All reagents used were of analytical grade. Betaine was purchased from Sigma-Aldrich (Chemie, Steinheim, Germany), as well as ammonium formate, sodium acetate, sodium hydrogen phosphate, acetonitrile, methanol and hydrochloride acid, which were used for eluent preparation and for extraction procedures. For the QuEChERS procedure, magnesium sulphate and sodium chloride were from UCT (Bristol, USA); PSA sorbent from J.T. Baker and graphitized black carbon from Supelco (Bellefonte, USA). High-purity water (18.2 MΩ cm resistivity at 25 °C), produced by a Milli-Q system (Millipore, El Passo, TX, USA), was used.

### Samples

The two different varieties of beetroot studied, *B. vulgaris* red and golden, were acquired from local markets. Each sample was peeled and the vegetable was ground in order to obtain a homogenized and soft pulp. Finally, the pulp was filtered in order to separate it from the juice. The samples thus prepared were stored in a freezer at −10 °C until analysis.

### Instrumentation

An Agilent 1100 liquid chromatograph (Agilent, Waldbronn, Germany), equipped with a diode-array detector and an Agilent 6410 Triple Quad mass spectrometer (Agilent, Waldbronn, Germany), was employed for analysis. Based on the high polarity of betaine, a hydrophilic interaction chromatography column (HILIC) was used for its analysis. A HILIC Kinetex column (100 mm × 4.6 mm, 2.6 μm) (Phenomenex, Torrance, CA, USA) and a mobile phase (flow rate 0.6 mL/min) composed of acetonitrile (A, 75% *v*/*v*) and 10 mM ammonium formate buffer (B, 25% *v*/*v*), acidified to pH 3 with formic acid, in isocratic elution mode were used. Injection volume was 5 μL. Data were collected with the use of Agilent Mass Hunter, software version B.04.01.

Electrospray ionization (ESI) was applied in the positive ion mode. Nitrogen was used in the ion source and the collision cell. Full-scan mass spectra were recorded within the mass range of *m*/*z* 50–500 Da. Additionally, multiple reaction monitoring (MRM) mode was applied for the quantitative analysis. Three main operation parameters of MS/MS detector, namely collision energy (CoE), fragmentor voltage (FV) and temperature of the source (Temp), were optimized by means of a Central Composite Design (see ‘Optimization of MS parameters through experimental design’ section).

### Optimization of MS parameters through experimental design

Design of experiments (DOE) is a useful approach for the optimization of the performance of a system with known variables. In this work, a central composite design (CCD) was used to estimate constant, linear terms, interactions between the different variables and quadratic terms [14], as indicated by the following model, in which in case of more than two variables the upper-level interactions are not taken into account):

$$ Y={b}_0+{b}_1{X}_1+{b}_2{X}_2+{b}_3{X}_3+\left({b}_{12}{X}_1{X}_2+{b}_{13}{X}_1{X}_3+{b}_{23}{X}_2{X}_3\right)+{b}_{11}{X}_1^2+{b}_{22}{X}_2^2+{b}_{33}{X}_3^2 $$

In this study, the response *Y*, was the betaine peak area while *X*_1_, *X*_2_ and *X*_3_ were CoE, FV and Temp, respectively. *b*_*i*_ are the coefficient of the linear term, *b*_*ij*_ are the coefficients of the interactions and *b*_*ii*_ are the coefficient of quadratic terms. Parameters levels and statistical data treatment are detailed in the ‘Optimization of HILIC-MS/MS conditions through DOE’ section.

### Betaine extraction

For the extraction and the purification of betaine from beetroot samples, different approaches were evaluated and compared. For liquid samples, such as the beetroot juice, SPE or micro extraction by packed sorbents (MEPS) were used, since they ensure extraction and clean-up in the same step. For the solid portions of *B. vulgaris* (peel and pulp), ASE was used for the extraction of betaine from the matrices, while SPE and MEPS procedures were used to clean-up betaine from the co-extracted species.

#### Solid-phase extraction

For the solid-phase extraction (SPE) procedure, the performance of three sorbents (3 mL, 500 mg) was compared. In detail, a strong cation exchanger resin, Bakerbond SCX aromatic sulfonic acid (Agilent), a mixed mode hydrophilic/lipophilic polymer based resin, OASIS HLB (Waters) and a silica gel sorbent, Discovery SPE Pure Silica (Supelco), were used.

Each cartridge was conditioned with different solutions, depending on the type of adsorbent: (i) SCX was conditioned with 3 mL of water, followed by 3 mL of water acidified to pH 2 with HCl; (ii) HLB with 3 mL of methanol followed by 3 mL of water, while (iii) pure silica with 5 mL of methanol.

For each cartridge, aliquots of 4-mL solutions containing 50 μg/L of betaine were loaded with a flow rate of 1–2 mL/min flow rate. The eluents tested in the recovery step were chosen according to the expected analyte–sorbent interactions. Before elution, each cartridge was washed with 2 mL of the proper solvent (H_2_O acidified to pH 0.5 for SCX cartridge, H_2_O for polymeric sorbent and methanol for pure silica resin) to remove unretained compounds. To check the retention of the analyte, the following fractions were collected during each step of the SPE protocol and then analysed by HPLC-MS/MS: (i) the solution after loading, (ii) the washing solution and (iii) the eluate.

#### Accelerated solvent extraction

Dionex ASE 200 (Sunnyvale, CA) accelerated solvent extractor, equipped with 33-mL stainless steel cells and 60-mL collection vials was used. Extraction was performed mixing roast sand with the beetroot peel or pulp samples; two different extraction solvents were used (methanol and water-methanol, 50:50% solution). 1 g of sample was mixed with 3 g of sand and 3 cycles of 5 min with 4 mL each of extraction solvent were performed. Based on the only other paper present in literature (in which however betaine was extracted from algae) the temperature applied was 50 °C [15]. After extraction, the extract was diluted 1:5000 with methanol and purified with the optimized SPE procedure, using pure silica cartridges (see below).

#### Micro extraction by packed sorbents

The evaluation of MEPS extraction/purification technique was performed using the sorbent which turned out to be the best one in terms of recovery during the previous SPE extraction of betaine. MEPS sorbent was a pure silica adsorbent placed in a cartridge, located in the needle and it was obtained from SGE Analytical (Melbourne, Australia).

The main steps of the MEPS extraction are as follows: (i) conditioning of the cartridge with 200 μL of methanol, (ii) loading of 50-μL sample in the syringe, (iii) removal of the needle and the cartridge, (iv) drying of the cartridge with air, (vi) loading of the eluent solution (50 μL) and (vii) removal, again, of needle and cartridge, pushing the solution in a vial for analysis.

#### QuEChERS

To propose a quick procedure to extract and isolate betaine that does not necessarily require two different purification approaches, a QuECheRS method was developed. In detail: 1 g of *B. vulgaris* peel was placed into a 50-mL centrifuge tube; 20 mL of water-methanol solution (1:1) were added, together with 4 g of MgSO_4_ and 1 g of NaCl. The tube was shaken for 1 min and next centrifuged at 6.000 rpm for 5 min (extraction step). Subsequently, 5 mL of the supernatant were transferred to a vial containing 150 mg PSA and 50 mg graphitized carbon black (GCB); the vial was shaken again for 1 min (clean-up step). The tube was centrifuged for 10 min, 10,000 rpm, to completely separate the dispersive phases. Finally, 1 mL of the supernatant was transferred to the vial for the chromatographic analysis.

For all the techniques evaluated, recoveries are expressed as the average of three independent extractions; in parallel, a blank was processed. Recovery for each protocol was assessed using HILIC-MS/MS technique.

#### Optimized extraction conditions

Details of the optimized extraction procedures for both solid (peel and pulp) and liquid beetroot matrices (juice) are summarized in Table 1.

**Table 1:** Extraction protocol optimized and applied for betaine determination in beetroot pulp, peel and juice

	Extraction step	Purification step
Liquid matrices (juice)	SPE ✓ Conditioning of pure silica cartridge = 5 mL CH_3_OH ✓ Loading 4 mL sample ✓ Washing of the cartridge = 5 mL CH_3_OH ✓ Elution = 4 mL H_2_O	
Solid matrices (peel, pulp)	ASE ✓ 1 g of sample mixed with 3 g of dispersive roast sand ✓ 3 cycles of 5 min with 4 mL CH_3_OH at 50 °C ✓ 1:5000 dilution of the extract with methanol	SPE ✓ Conditioning of silica cartridge = 5 mL CH_3_OH ✓ Loading 4 mL sample ✓ Washing of the cartridge = 5 mL CH_3_OH ✓ Elution = 4 mL H_2_O

## Results and discussion

### Optimization of HILIC-MS/MS conditions through DOE

Due to the polarity of betaine, the HILIC mode was selected for the analysis of the analyte. For the separation, silica gel was used as the most commonly used stationary phase in HILIC mode. Betaine was expected to be retained on the stationary phase due to polar interactions, e.g. hydrogen bonding and electrostatic interactions. In order to improve peak symmetry and to increase sensitivity, a buffer was introduced in the mobile phase. The final mobile phase composition consisted of 75% *v*/*v* of acetonitrile and 25% *v*/*v* of 10 mM ammonium formate buffer (pH 3). Acetonitrile-rich mobile phase, as known, are beneficial for MS sensitivity. Despite the slight peak width broadening observed with acid eluents, acid conditions were preferred to enhance MS sensitivity in the positive ion mode. Under the above-mentioned elution condition, the analysis of betaine can be performed within 6 min.

A full-scan mass spectrum of betaine was recorded obtaining the two parent ions at *m*/*z* 118.1 [M + H]^+^ and *m*/*z* 235.1 [2 M + H]^+^, corresponding to the molecular mass of betaine (117 Da). Additionally, one signal was observed for product ion spectra at *m*/*z* = 58.1 Da. On this basis, the transition 118.1 → 58.1 Da was used to quantify betaine in the MRM mode. Since it is well established that the MRM sensitivity depends drastically on the tuning of instrument parameters [16], an optimization of collision energy (CoE), fragmentor voltage (FV) and temperature of the source (Temp) was performed by means of a CCD. The response variable was the betaine MRM peak area. It is worth to be mentioned it is well known that the expected concentrations of betaine in beetroots samples are not in trace levels. However, the effort spent in optimizing the MS/MS parameters by the experimental design technique is justified by possibility to apply this chromatographic method also to other applications, quantifying matrices with trace amounts of betaine (which are several, as listed in the work of Slow et al. [17]).

To investigate the effect of each of the above-mentioned factors and to subsequently optimize the response, the (−1) and (+1) levels were the following: CoE 2 and 40 eV; FV 105 and 165 V and Temp 235 and 345 °C. At the central level (30 eV, 85 V and 55 °C), three replicates were performed.

**Fig. 2:**
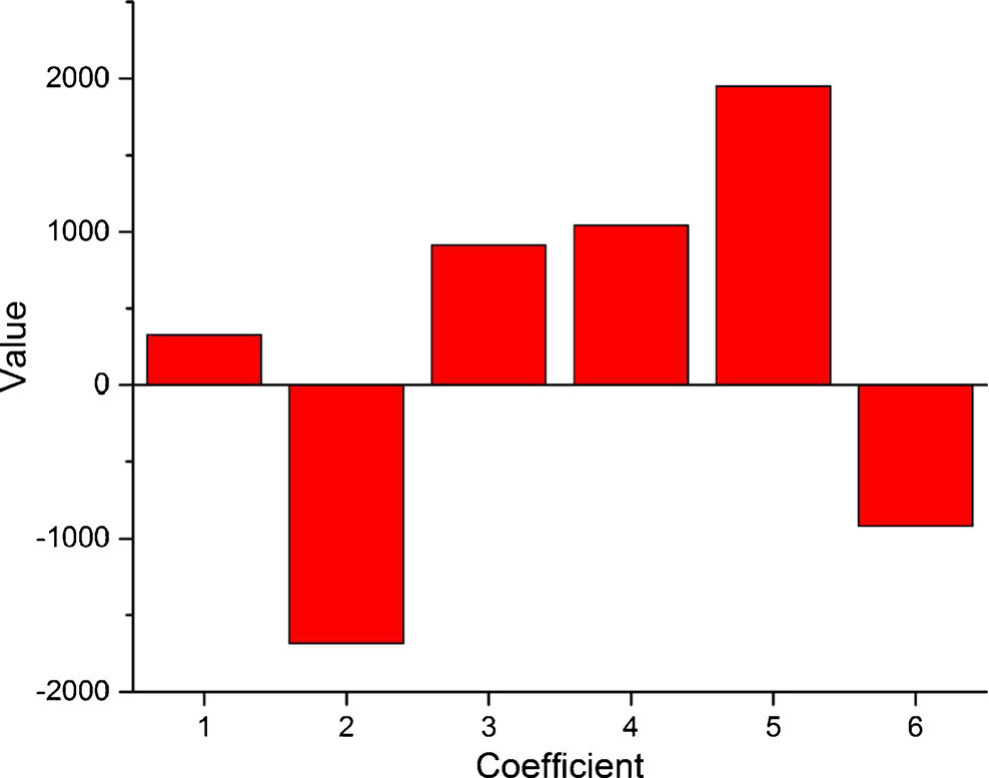
Graphical representation of the coefficients obtained for the optimization of MS/MS detector parameters. *1*, *2* and *3* (corresponding to *b*_1_, *b*_2_ and *b*_3_) refer to linear terms of CoE, FV and Temp, respectively, while *4*, *5* and *6* (corresponding to *b*_11_, *b*_22_, *b*_33_) refer to quadratic ones

**Fig. 3:**
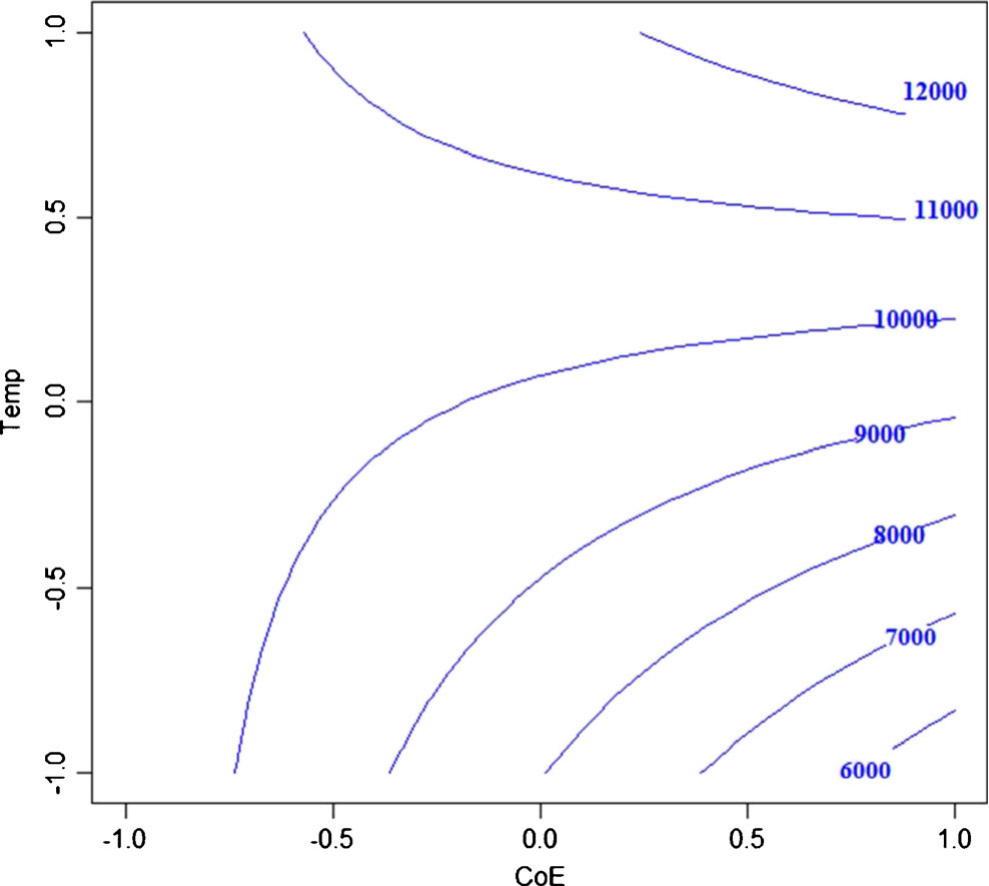
Graphical representation of peak area as a function of collision energy and temperature of the Source at FV = 105 V

Main and quadratic effects of the variables considered were calculated with Yates algorithm and R-chemometric software. A graphical representation of the *b*_*i*_ and *b*_*ii*_ coefficients is represented in Fig. 2. As shown, the linear term *X*_2_ (FV) suggests that this parameter strongly influences the response. In detail the increase of FV causes a decrease of the response. On the contrary, the increase of the temperature of source leads to a higher response. The CoE parameter only slightly influences the sensitivity for betaine. However, all the quadratic terms (from 4 to 6, corresponding to *b*_*ii*_ coefficients) have high values, meaning that the dependence of the response on parameters is not linear, as confirmed by the response surfaces graphs (Fig. 3).

Following the assumption that FV has a negative correlation with MRM peak area (higher values means lower sensitivity) and that using too low FV values fragmentation is not possible, the response graph was obtained keeping constant FV to its lower coded value −1 (corresponding to 105 V). Lines in Fig. 3 represent the iso-curves, which contain all the experimental conditions providing the same response. As shown, experimental conditions that lead to the highest peak area can be easily derived. The following conditions CoE = 40 eV, FV = 105 V and Temp = 345 °C were considered optimal and experimentally tested. The results obtained showed an increase of about 186% of betaine peak area if compared to initial conditions, thus confirming the hypothesis derived by the DOE.

### Validation of HILIC-MS/MS technique

Validation of the HILIC-MS/MS technique was performed investigating the main performance parameters of analytical method validation suggested by European Union (EUR-FA Guide, Annex I) and IUPAC guidelines [18], such as linearity, limits of detection, limits of quantification and instrument reproducibility.

The calibration curve for betaine was linear over the concentration range 5–500 μg/L. Standard curve used in this study was determined using the following linear regression: *y* = 239.02*x* − 19.45 (*R* ^2^ = 0.99992).

The linearity of the HILIC-MS/MS method was verified over two orders of magnitude with root mean square error (RMSE) for calibration equal to 65.80 for HILIC (related to chromatographic peak areas of 10^4^/10^5^ order of magnitude, respectively). Limit of detection (LOD) and limit of quantification (LOQ) were evaluated as LOD = 3 × SD_xy_/*b* and LOQ = 10 × SD_xy_/*b* (where SD_xy_ is the standard deviation of the response and *b* is the slope of the calibration curve) [19]. There were found to be 0.95 and 2.87 μg/L, respectively.

The detection limits obtained for the HPLC-MS/MS in HILIC mode are lower (about 10 times) if compared with the ones obtained in literature [7, 20] using the same detector, mainly due to the CCD optimization of the main detector parameters. It should be remarked that very few approaches for determination of betaine by detectors different from MS are available. Shin et al. [8] propose betaine determination by evaporative light scattering detection at concentration levels higher than 1 mg/L, three orders of magnitude higher than the approach here proposed.

Inter- and intra-day reproducibility of the instrument (expressed as relative standard deviation, % RSD) were evaluated both for retention times and peak areas. A standard solution of betaine (5 μg/L) was injected repeatedly in the same day and for 2 weeks: the intra-day RSD obtained was 1.4% for retention times and 2.1% for the peak area, while the inter-day values were 2.8 and 4.1%, respectively, supporting the robustness of the chromatographic technique.

### Optimization of the extraction protocol

In order to extract betaine from beetroot samples and to isolate the molecule from interfering species, an extraction procedure was optimized comparing the performance of different approaches. In previous studies showing the determination of betaine in numerous food and vegetable matrices, solid samples were simply mixed with water, homogenized and centrifuged to recover the aqueous supernatant. The aqueous supernatant was then extracted with dichloromethane which removes hydrophobic compounds without removing betaine. Also, in case of liquid samples, they were shaken with an equal volume of dichloromethane, centrifuged and the resulting aqueous layer directly employed for analysis [17, 21]. However, these procedures are not selective towards betaine, and high hydrophilic and polar compounds are dissolved in the aqueous phase which is injected and analysed. As well, described by Wruss et al., beetroots and their derivative products (as for examples juices) have high concentration of sugars (glucose, fructose and sucrose, from 1.5 to 73.5 g/L for 15 mL of homogenized sample) and betalains that serve as colour pigments (mean concentration of 1.1 g/L). All the above-mentioned compounds are possible interfering species that should be removed and, therefore, the optimization of purification method to isolate betaine is discussed in the next paragraphs.

As previously mentioned, betaine could be present in both liquid (juice) and solid (peel and pulp) matrices. For the latter, extraction and clean-up steps are necessary, while for liquid samples, both the steps could be done within the same technique.

#### Extraction of betaine from liquid matrices

An SPE procedure was optimized comparing the performance of three different sorbents, see Table 1. As shown, all the tested sorbents interact with the analyte; retention was quantitative for SCX and pure silica cartridges, while a decreased but still significant retention of betaine was observed for the HLB sorbent (75%). The analysis of the washing solutions, before the elution step, showed no evidence of betaine. Since we observed that when using pure silica cartridge, betaine has to be dissolved in methanol; otherwise, interactions with sorbent are weak and low recoveries are obtained, this solvent was used during the loading step. This feature must be taken into account also for the selection of the extraction solvent for the ASE procedure (see ‘Extraction of betaine from solid matrices’ section).

For the elution of the betaine, different solvents (compatible with the HILIC-MS/MS analysis) were tested, depending on the physico-chemical characteristics of the sorbent and on the hypothesised interactions. Aromatic sulfonic acid groups present in the SCX interact with the positively charged amino group of betaine by electrostatic interactions. Consequently, eluents that can give rise to competitive interaction were evaluated. 1 M HCl and three different buffers, formate, acetate and phosphate, at different pH (2, 4, 8, respectively) at increasing concentrations (20, 50, 100 mM) were tested. At the tested conditions, elution recoveries ranged from 5 to 10%.

The elution from the polymeric OASIS HLB cartridge was evaluated with 4 mL of acetonitrile and a solution 50:50 *v*/*v* acetonitrile/water or water, which were not effective in the recovery of betaine.

The expected interaction between the silica sorbent and betaine is based on polarity and adsorption on hydrogen bonding. For the elution of betaine from silica, 2 aliquots of 2 mL of water were used for recovery, finally reaching a quantitative release of the analyte.

Since the procedure for extraction and recovery of betaine appeared to be very easy, cheap and effective, the same adsorbent and elution conditions were selected for MEPS technique as well. MEPS procedure has lower costs than SPE because volumes involved are typically very small. However, the enrichment factor is not as high as in other methods [22]. In our MEPS procedure, approximately 1 mg of solid packing material was inserted into a syringe (250 μL) as a plug with a filter on both sides. The same elution solvent successfully applied for SPE was used in case of MEPS. The effect of the sample volume loaded on MEPS substrate (50–100 μL) on recovery was initially evaluated. Results in Table 2 show that, at the optimal volume (50 μL), the obtained retention is about 90%, while the elution is quantitative. If compared to the quantitative recovery obtained with SPE, this decrease could be ascribed to the lower amount of sorbent used in this technique (1 mg in MEPS vs 500 mg in SPE).

**Table 2:** Recovery yields obtained for each tested substrate. SPE conditions: Sample: 50 μg/L betaine in 4 mL of water solution; elution volume: 4 mL. MEPS conditions: 10 μg/L betaine in 50 μL water solutions; elution volume: 50 μL. For activation and recovery procedures, see text

	Cartridge	Retention [%]	Elution [%]
SPE	J. T. Baker SCX	Quantitative	10%^a^
	Waters OASIS HLB	75.4 ± 4.2%	9.3 ± 2.1%^b^
	Supelco PureSilica	Quantitative	Quantitative^c^
MEPS	Pure Silica	89.3 ± 2.1%	Quantitative^d^

Elution solvents: ^a^1 M HCl, ^b^Water, ^c^Water, ^d^Water

##### Analysis of betaine from juice

In order to evaluate the efficiency of the SPE technique on real matrices, the procedure was used for the determination of betaine in beetroot juice. The study has been performed on both the red and golden beetroot juices. Recoveries were 92.7% (red) and 93.3% (golden), with high accuracy and repeatability (RSD <1.2%). Lower recovery values, compared to standard solution samples, could be ascribed to competition effects that occurred between the matrix of the beetroot juice and the silica sorbent. The same extraction was performed with optimized MEPS procedure, which confirmed slightly lower recoveries (93%) than SPE.

**Fig. 4:**
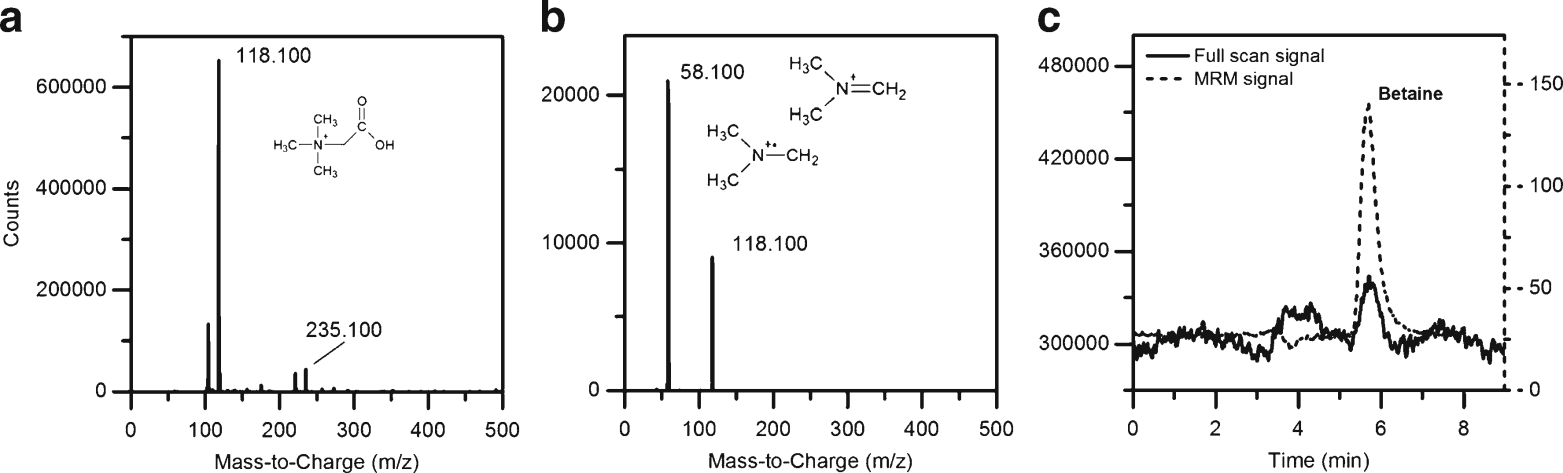
Analysis of the eluted fraction after SPE extraction of betaine from *Beta vulgaris* golden juice. Betaine full-scan spectrum and product ion spectrum (**a** and **b**, respectively), and chromatogram overlay of the betaine signal obtained both in MRM and full-scan mode (**c**). Instrumental conditions are detailed in the ‘Instrumentation’ section

In Fig. 4, typical chromatograms obtained from the analysis of a *B. vulgaris* golden juice are shown. Full scan (Fig. 4A) and product ion spectra (Fig. 4B) exhibit typical pattern of betaine (see ‘Optimization of HILIC-MS/MS conditions through DOE’ section). The chromatograms obtained both in full scan and MRM detection are overlaid in Fig. 4C.

#### Extraction of betaine from solid matrices

Since it was not possible to evaluate the extraction efficiency on a blank solid sample, tests were performed comparing betaine concentration in pulp and peel of red and golden beetroots with the concentration of spiked samples.

An ASE procedure was used, comparing the performance of two different extraction solvents: a methanol and a solution of water-methanol 50:50% *v*/*v*. Even if both solvents lead to extraction yields of about 95%, methanol was selected since it is fully compatible with subsequent SPE step using pure silica cartridge (as previously optimized and described in the ‘Extraction of betaine from liquid matrices’ section). For both pulp and peel matrices, high recoveries (from 94.4% of red beetroot’s peel to 96.5% of red beetroots pulp, RSD <1.05%) were achieved, confirming a high reproducibility and accuracy of the procedure. To the best of our knowledge, this is the first time that an ASE procedure is proposed for the extraction of betaine from beetroots.

Details of the optimized extraction procedure are summarized in Table 1, see ‘Optimized extraction conditions’ section, together with the ones optimized for liquid matrices. The overall ASE/SPE procedure takes advantages of both the high-pressure conditions of ASE, which in turn provide faster extraction times than traditional extraction apparatus [9] (12 min against 3 h), and of the higher safety conditions of SPE purification which is based on water and methanol, rather than on more toxic organic solvents (e.g. dichloromethane [10]).

### Evaluation of betaine content in *B. vulgaris*

Once optimized, the procedure for the extraction of betaine from both the solid and the liquid parts of the beetroots, a quantitative determination of this compound was performed in real samples. In literature, many studies determine the content of betaine in foods, especially in sugar beet and molasses, but this is the first time in which betaine content is specifically quantified for each portion of *B. vulgaris* red and gold.

**Fig. 5:**
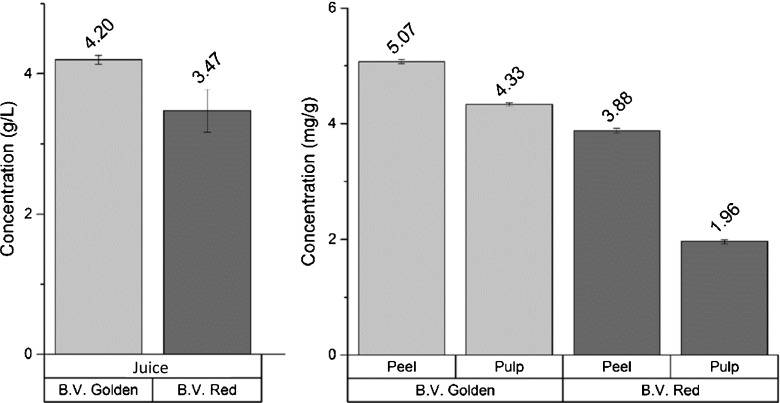
Comparison of the betaine concentration level in all the three portions (juice, peel and pulp) of *Beta vulgaris* red and golden. Extraction procedure is summarized in Table 2 and ‘Experimental section’

Figure 5 summarizes the concentration levels of betaine found in each portion of beetroots, both for red and gold vegetable. It can be observed that *B. vulgaris* golden variety has the highest concentrations of betaine in juice, pulp and peel. However, both the varieties are generally characterized by high levels of betaine (from 2 to 5 mg/g).

Data have been compared with determinations already performed on sugar beets and on different species of the same family of *B. vulgaris* (Chenopodiaceae). It is interesting to highlight that *B. vulgaris* species have higher concentrations of betaine if compared with other entities that belong to the same family. As an example, betaine concentration is typically about 0.2–0.3% (2–3 mg/g) also in sugar beets [23], while in fresh spinach, which belongs to the same family, betaine is present at concentration 5 to 10 folds lower [10].

### QuEChERS procedure

The extraction procedures optimized in the previous paragraphs allow us to extract betaine from all portions of *B. vulgaris* vegetables. However, for solid samples (as peel and pulp), this extraction is accomplished by two different, consecutives techniques: ASE for extraction and SPE for isolation of betaine. In order to obtain betaine extraction by unique approach, a QuEChERS procedure for the extraction of betaine from *B. vulgaris* samples was here evaluated for the first time.

The QuEChERS technique, developed for the extraction of pesticides from food [24], with extensive applications event to other analytes and matrices [25, 26] is an easy and fast procedure, since it is based on liquid extraction of target analytes, assisted by hand shaking and followed by a dispersive-SPE (d-SPE) clean-up step, using selective adsorbents.

A modified QuEChERS procedure was here tested on the peel of golden beetroot: this portion was chosen since, according to the previous results, it has the highest concentration of betaine. In spite of acetonitrile, used in the traditional procedure, methanol was selected as the extraction solvent, due to the high affinity towards betaine observed in the extraction techniques previously commented. Magnesium sulphate and sodium chloride salts were added in order to enhance the salting-out effect, promoting a better extraction of betaine. For the d-SPE step, first tests were performed using a combination of PSA and graphitized carbon black (GCB) resins. PSA is necessary to remove co-extracted sugars and weak organic acids, while GCB is necessary to remove dyes and aromatic interferences. Results obtained after HILIC-MS/MS analysis showed that a recovery of 75.3 ± 0.8% was obtained. Lower recoveries than the ASE/SPE procedure could be explained by betaine interaction with the GCB sorbent. In order to confirm the hypothesis, a betaine solution was prepared at the same concentration present in Golden beetroot peel and was put in contact with 50 mg of GCB, shaken for 1 min, centrifuged and analysed, obtaining an adsorption of betaine of 25.2%.

It should be mentioned that the replacement of GCB with a C18 resin was not effective in removing co-extracted dyes and additional purification would be necessary, losing the intrinsic advantages of QuEChERS technique.

In conclusion, the method detection limits (MDLs) obtained for betaine extraction from *B. vulgaris* peel by ASE/SPE and QuEChERS were found to be 4.1 (93%) and 11.86 (75%) μg/kg, respectively. Despite the lower recoveries of QuEChERS if compared to the ASE/SPE procedure, and slightly higher MDLs, the ease of the QuEChERS technique, which can be applied without the SPE purification procedure, can represent a positive improvement. Moreover, if compared to ASE/SPE, QuEChERS procedure is also time-saving (as described in the ‘QuEChERS’ section, since only 1 min of shaking is needed for the extraction and the d-SPE steps) and far cheaper in terms of laboratory equipment required (plastic tubes and a centrifuge system) than the ASE system.

## Conclusions

This work presents the first report on the study and optimization of the extraction and determination of betaine from two different *B. vulgaris* varieties, red and gold. The innovative coupling of the optimized ASE and SPE techniques allows to successfully isolate betaine from complex matrices such as liquid and solid portions of beetroot. The solvents used for both the procedures are fully compatible with the following steps of the analytical method, avoiding reconstruction and evaporation steps which could reduce the recoveries of the overall method. Central composite design was applied for the optimization of a HILIC-MS/MS technique with better sensitivity than previously published works. Excellent extraction performances were achieved in terms of method sensitivity and robustness with recoveries in real samples as high as 93%.

Moreover, for the first time, a QuEChERS procedure was successfully tested: although recoveries achieved were slightly lower than ASE/SPE procedure (recoveries about 75%), the high reproducibility and ease, this approach can justify its use for a quantification of betaine in complexes samples, such as *B. vulgaris*.

Finally, a quantification of betaine in red and golden *B. vulgaris* varieties and in each portion of them was performed, obtaining high concentrations, especially in golden *B. vulgaris*, supporting the possible administering of these vegetables and their juice in diets of patients that exhibit deficiency of this compound.
